# Cardiovascular Disease Epidemiology in Portuguese-Speaking Countries: data from the Global Burden of Disease, 1990 to 2016

**DOI:** 10.5935/abc.20180098

**Published:** 2018-07

**Authors:** Bruno Ramos Nascimento, Luisa Campos Caldeira Brant, Gláucia Maria Moraes de Oliveira, Marcus Vinícius Bolívar Malachias, Gabriel Moreira Alves Reis, Renato Azeredo Teixeira, Deborah Carvalho Malta, Elisabeth França, Maria de Fátima Marinho Souza, Gregory A. Roth, Antonio Luiz P. Ribeiro

**Affiliations:** 1 Universidade Federal de Minas Gerais (UFMG), Belo Horizonte, MG - Brazil; 2 Universidade Federal do Rio de Janeiro, Rio de Janeiro, RJ - Brazil; 3 Faculdade Ciências Médicas de Minas Gerais, Belo Horizonte, MG - Brazil; 4 Ministério da Saúde, Brasília, DF - Brazil; 5 University of Washington, Seattle, WA - USA

**Keywords:** Cardiovascular Diseases, Epidemiology, Mortality, Global Burden of Disease / trends

## Abstract

**Background:**

Portuguese-speaking countries (PSC) share the influence of the Portuguese culture but have socioeconomic development patterns that differ from that of Portugal.

**Objective:**

To describe trends in cardiovascular disease (CVD) morbidity and mortality in the PSC between 1990 and 2016, stratified by sex, and their association with the respective sociodemographic indexes (SDI).

**Methods:**

This study used the Global Burden of Disease (GBD) 2016 data and methodology. Data collection followed international standards for death certification, through information systems on vital statistics and mortality surveillance, surveys, and hospital registries. Techniques were used to standardize causes of death by the direct method, as were corrections for underreporting of deaths and garbage codes. To determine the number of deaths due to each cause, the CODEm (Cause of Death Ensemble Model) algorithm was applied. Disability-adjusted life years (DALYs) and SDI (income per capita, educational attainment and total fertility rate) were estimated for each country. A p-value <0.05 was considered significant.

**Results:**

There are large differences, mainly related to socioeconomic conditions, in the relative impact of CVD burden in PSC. Among CVD, ischemic heart disease was the leading cause of death in all PSC in 2016, except for Mozambique and Sao Tome and Principe, where cerebrovascular diseases have supplanted it. The most relevant attributable risk factors for CVD among all PSC are hypertension and dietary factors.

**Conclusion:**

Collaboration among PSC may allow successful experiences in combating CVD to be shared between those countries.

## Introduction

Cardiovascular diseases (CVD) are a major cause of death worldwide. Although CVD are not the first cause of death in many low- and middle-income countries, they account for 80% of the deaths and 88% of the premature deaths in those countries.^1^ The control of infectious, maternal and child diseases, the increase in life expectancy and the growing urbanization have contributed to the trend towards increasing the importance of CVD in low- and middle-income countries. The implementation of health policies, such as promotion of a healthy lifestyle, access to primary and secondary prevention of CVD and treatment of acute cardiovascular events, is, thus, essential for CVD control in all countries.^1^

Portuguese-speaking countries (PSC) were culturally influenced by Portugal at different levels.^2^ The study of the trends in morbidity and mortality from CVD in those countries can provide us useful data regarding the similarities and differences between them. Those data can provide an exchange of information between countries regarding well-succeeded actions for fighting CVD, in addition to allowing reflections on the influence of culture on the burden of CVD.

The "Global Burden of Disease Study" (GBD Study) is an important epidemiological observational study that uses metrics of morbidity and mortality related to major diseases, injuries and risk factors at global, at national and regional levels. One of the GBD Study objectives is to understand, by assessing the trends, the changes in the profile of the diseases that affect the 21st century populations.^3^

This study was aimed at describing the trends of CVD morbidity and mortality in the PSC between 1990 and 2016, based on the estimates of the GBD 2016 Study, and to assess the association between those trends and the sociodemographic index (SDI) of those countries.

## Methods

### Portuguese-speaking countries

**Figure 1:**
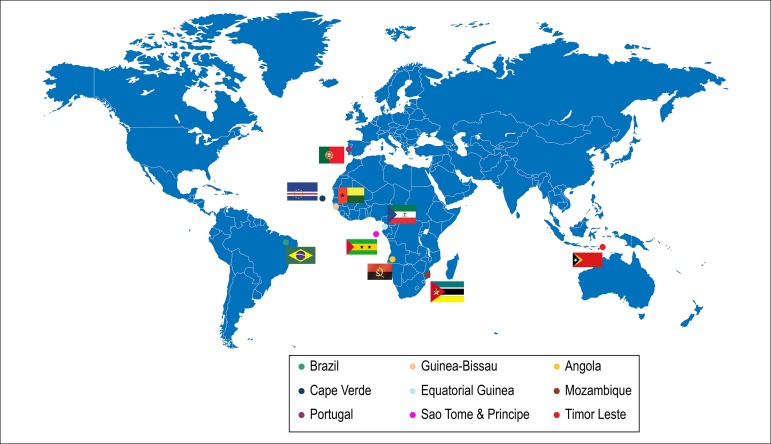
Global map showing the location of the Portuguese-speaking countries, 2017.

The PSC are the official members of the Community of Portuguese-Speaking Countries (CPLP), an international organization created in July 17, 1996, aimed at "deepening the mutual friendship and cooperation between its members".^2^ The initial list of countries included Angola, Brazil, Cape Verde, Guinea-Bissau, Mozambique, Portugal and Sao Tome and Principe. In 2002, after its independence, Timor-Leste was accepted as a member. In 2014, Equatorial Guinea was accepted as a member, after Portuguese was adopted as the official language of the country. Table 1 and Figure 1 show the location and the demographic, social and economic characteristics of the PSC.

**Table 1:** Demographic, social and economic characteristics of the Portuguese-speaking countries, 2013

	Angola	Brazil	Cape Verde	Equatorial Guinea	Guinea-Bissau	Mozambique	Portugal	Sao Tome and Principe	Timor-Leste
Population (millions)	19	201	1	1	2	24	10	0.2	1
Population density (inhab/km²)	15	24	127	39	44	31	113	190	79
Area (1,000 km²)	1247	8516	4	28	36	786	92	1.0	15
GDP (USD, billions)	125	2473	2	22	1	16	226	0.3	1
GDP per capita (USD)	4805	12217	3589	20247	611	606	21619	1620	1108
Major religion	Catholicism	Catholicism	Catholicism	Catholicism	Islam	Catholicism	Catholicism	Catholicism	Catholicism
Public expenditure with health (%GDP)	2.6	4.0	2.5	2.6	1.1	2.1	9.1	2.0	10.4
Public expenditure with education (%GDP)	4.9	6.2	5.5	2.2	2.2	5.7	4.2	5.6	16.2
Illiteracy rate (% population)	28.4	8.5	12.8	6.0	40.1	41.2	5.2	9.6	35.9
Sociodemographic index*	0.42	0.66	0.55	0.61	0.29	0.28	0.75	0.45	0.45

* Data from 2015. GDP: gross domestic product. USD: United States dollars.

### The GBD Study

The GBD Study is a multinational collaborative research project with the goal of producing consistent estimates of health loss due to over 333 diseases and injuries. A wide range of data sources (data of national surveillance, verbal autopsy and vital registration, published and unpublished registries of diseases, and published scientific literature) and methods are applyed to produce specific results with uncertainty intervals for age, sex and country for the years 1990-2016, and such results are annually updated for the entire temporal series. The present study is based on data from the GBD 2016 Study and its previously detailed methodology.^4-6^

### Definitions of CVD

The nine most common global causes of death and morbidity related to CVD and an additional category for 'other CVD' were considered, in addition to the estimates of global morbidity and mortality grouped by CVD.^7^ The underlying cause of death was defined as CVD according to the International Classification of Diseases (ICD) codes, from the death certificate (DC), the basic document that informs the causes of death in countries with systems of vital statistics, such as Brazil. The following causes were analyzed, with their corresponding ICD-10 codes according to the GBD Study's classification list of causes: 1- rheumatic fever with cardiac involvement (codes I01-I01.9, I02.0, I05-I09.9); 2-ischemic heart disease (codes I20-I25.9); 3- cerebrovascular disease (CbVD) (G45-G46.8, I60-I69.9); 4- hypertensive heart disease (I11); 5- cardiomyopathy and myocarditis (A39.52, B33.2-B33.24, D86.85, I40-I43.9, I51.4-I51.5); 6- atrial fibrillation and flutter (I48); 7- aortic aneurysm (I71); 8- peripheral artery disease (I70.2-I70.7, I73-I73.9); 9-endocarditis (A39.51, I33-I33.9, I38-I39.9). Garbage codes, such as heart failure (I50) and pulmonary embolism (I26), which do not define the pathology that caused the death, were redistributed to those specific causes based on the GBD methodology, according to algorithms defined in that study.

Regarding mortality, those causes were grouped based on the specific sequelae of disease (for example, ischemic heart disease due to acute coronary syndrome, chronic stable angina, chronic ischemic heart disease and ischemic cardiomyopathy). Adjustments were performed for data that did not comply with the specific definition of case (for example, electronic confirmation for the clinical diagnosis).^4,8,9^

### Statistical analysis

The statistical models of the GBD 2016 Study previously reported were used.^4-6^ The source of data for the models are available on-line at Global Health Data Exchange (http://ghdx.healthdata.org/).

### Metrics of mortality and prevalence

The GBD 2016 Study used data available on causes of death in 195 countries. The information was collected according to international standards of death certification, using of information systems on vital statistics, mortality surveillance systems, survey, hospital registries and police registries.^4^ The data sources have regional particularities, such as in Brazil, where data were mainly obtained from the Mortality Information System (SIM) of the Brazilian Health Ministry, by using an automated coding system.^10^ In Brazil, all deaths require a medical DC provided by the attending physician. For the deaths occurring outside a healthcare facility, the causes are verified by the Death Verification Service, or by a civil agent when a physician is not available, and, in such cases, the causes of death are not registered.^11^ Deaths due to external causes are identified by a medical examiner at the Forensic Pathologist Service.

In addition, techniques to correct quality problems regarding the information about the causes of death were used.^9^ Corrections were made for underreporting of deaths and for causes considered not useful for the public health analysis, known as Garbage Codes. That term is used to describe causes that cannot or should not be considered as underlying causes of death, or that are unspecified within larger groups of causes. Algorithms of redistribution of Garbage Codes were developed by the GBD Study to increase the validity of the estimates. For this redistribution into specific causes of death, evidence from several sources, such as medical literature, expert opinion and statistical techniques, was considered.^4,8,12^

After treatment of data quality, the GBD 2016 Study used a variety of statistical models to determine the number of deaths per each cause, mainly by use of the Cause of Death Ensemble Model (CODEm) algorithm. To ensure that the number of deaths per cause does not exceed the total estimated number of deaths, a correction technique (CoCorrect) was used. Adjustment by this technique ensures that the sum of the estimated number of deaths per each cause does not exceed 100% of the estimated deaths in a certain year.^13^

The disease prevalence was estimated at a more detailed level of specific sequelae of disease, using as entry data the published systematic reviews of the scientific literature, as well as unpublished data of administrative registries and databases of the health system. Regression equations were used for data adjustment to define the standard case. The data presented were analyzed for the period from 1990 to 2016, and all analyses were stratified by sex and presented as absolute and age-standardized estimates, for the different PSC.

### Metrics of burden of disease

The disability-adjusted life years (DALYs) combine information regarding premature death (years of life lost: YLLs) and disability caused by the condition (years lived with disability: YLDs) to provide a brief measure of the healthy years lost due to the condition. The YLLs were calculated by multiplying the deaths observed at each specific age in one year of interest by the reference age-specific life expectancy estimated by use of life table methods. The YLDs were calculated by multiplying disease prevalence (in number of cases/year) by a health-state-specific disability weight representing a degree of lost functional capacity. The process of estimating the burden of the disability has been previously described in details.^6^ Briefly, the burdens of disability were determined via home interviews in several countries, in which the participants were asked to choose between lay descriptions of different health states.^14,15^ Adjustment for comorbidity was performed, simulating 40,000 individuals in each age-sex-country-year stratum exposed to the independent likelihood of developing each condition, based on the disease prevalence, with 95% uncertainty intervals (95% UI) reported for each estimate. Age-standardization was obtained via the direct method, applying a global age structure.

### Sociodemographic index

The SDI is used as an estimate of the socioeconomic level of each country to assess its association with the CVD burden, as a function of global epidemiological transition.^4,8^ Similarly to the method used to calculate the Human Development Index, the SDI was calculated for each country or territory from 1990 to 2016. The SDI is the weighted geometric mean of income per capita, educational attainment and total fertility rate, and allows the comparison of the performance of each country to those of countries with a similar socioeconomic level.

The SPSS software, version 22.0 for Mac OSX (*SPSS Inc., Chicago, Illinois*), was used to perform the correlation (Spearman correlation) between the country's SDI and the variation of the age-standardized mortality rates from CVD between 1990 and 2016. A p-value < 0.05 was considered statistically significant.

### Ethical considerations

This study was conducted in a public secondary database, without nominal identification, according to Decree 7.724, of May 16, 2012, and the Resolution 510, of April 7, 2016. The Brazilian GBD 2015 Study was approved by the Committee in Ethics and Research of the Minas Gerais Federal University (Project CAAE 62803316.7.0000.5149).

## Results

### Causes of mortality from CVD

**Figure 2:**
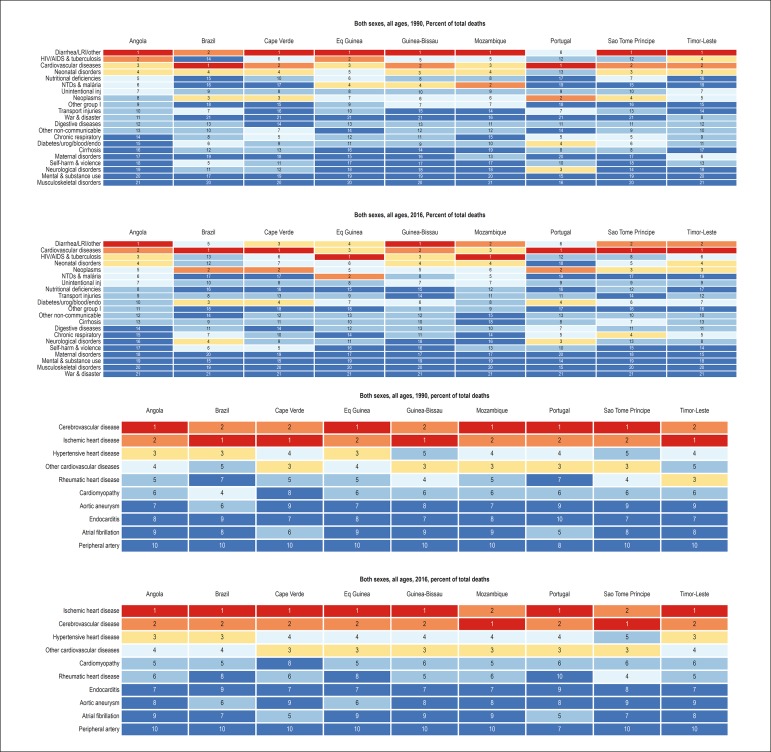
Most common causes of death, considering age-standardized mortality, in the Portuguese-speaking countries in 1990 and 2016. A: All causes; B: From cardiovascular diseases. LRI = lower respiratory tract infection; NTD = neglected tropical diseases.

The importance of CVD as the cause of death has increased in the PSC. In 1990, CVD were the main cause of death only in Brazil and Portugal, while, in the other countries, infectious diseases, such as diarrhea and respiratory infections, were the leading causes. In 2016, however, CVD became the leading cause of death in Cape Verde, Sao Tome and Principe and Timor-Leste, and ranked higher or maintained their ranks in the other countries (Figure 2A). Considering the causes of CVD, there was an increase in the proportional mortality from ischemic heart disease, which, in 2016, was the first cause of death in most countries studied, except for Mozambique and Sao Tome and Principe. In general, there was a reduction in proportional mortality from rheumatic heart disease (Figure 2B).

### Trends in the mortality rates from CVD between 1990 and 2016

**Figure 3:**
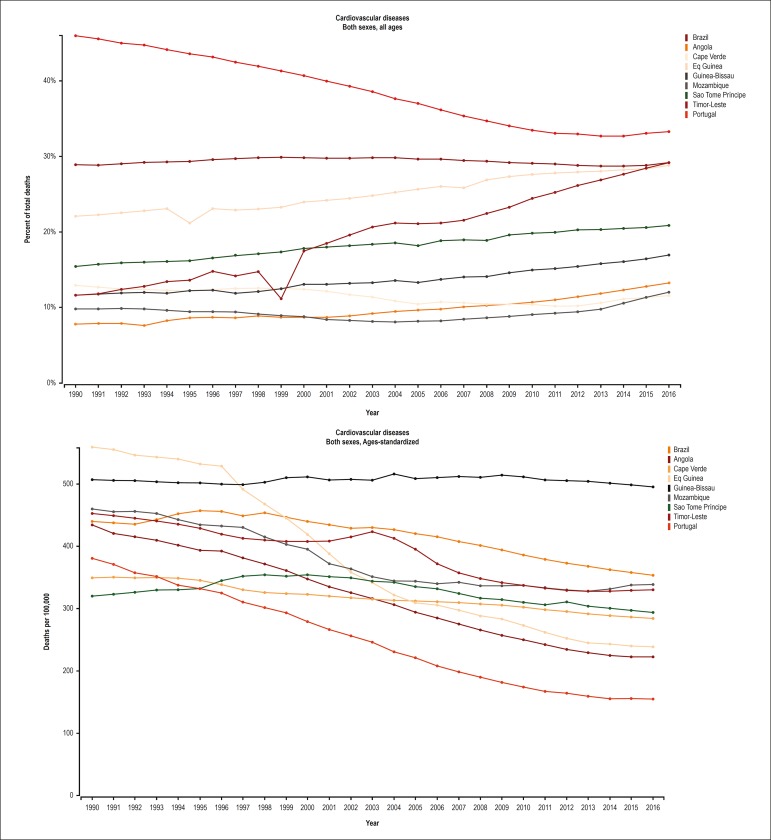
Mortality attributed to cardiovascular diseases in the Portuguese-speaking countries from 1990 to 2016. A. Proportional mortality from cardiovascular diseases (%), B. Age-standardized mortality rate from cardiovascular diseases (deaths/100,000).

Figure 3 shows an important reduction in proportional mortality from CVD and in the age-standardized mortality rate from CVD in Portugal, revealing that the decline in mortality occurred at all age groups. In Brazil and in Equatorial Guinea, the proportion of deaths from CVD remained stable, while a consistent reduction was observed over the past 15 years in the age-standardized mortality rate, suggesting there was mainly a reduction in premature mortality from CVD. In the other countries, the proportion of deaths due to CVD increased, and the reduction in age-standardized mortality rate from CVD declined less expressively, suggesting an increasing impact of CVD in those countries.

**Figure 4:**
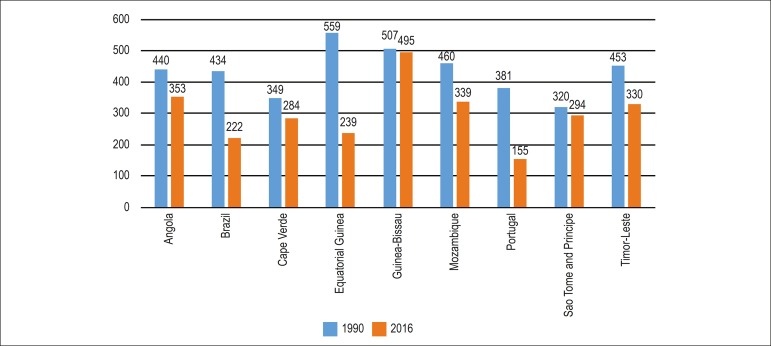
Age-standardized mortality rate in the Portuguese-speaking countries in 1990 and 2016.

Although the proportional mortality from CVD decreased in the PSC from 1990 to 2016, the decline was heterogeneous among the countries. Figure 4 shows the age-standardized mortality rates for each PSC in 1990 and 2016.

**Figure 5:**
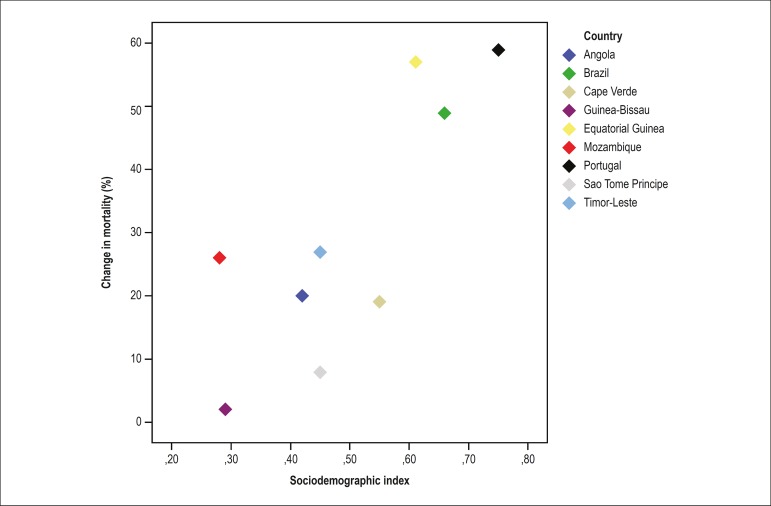
Correlation between the change in mortality from cardiovascular diseases between 1990 and 2016 and the sociodemographic index (r_s_ = 0.7; p = 0.04).

Figure 5 reveals the positive correlation between the reduction in age-standardized mortality rates from CVD between 1990 and 2016 and the SDI of the country (r_s_ = 0.7; p = 0.04), suggesting a reduction in mortality from CVD follows the improvement in the local socioeconomic conditions of the PSC.

### Lost of healthy life years (DALY) due to CVD

**Figure 6:**
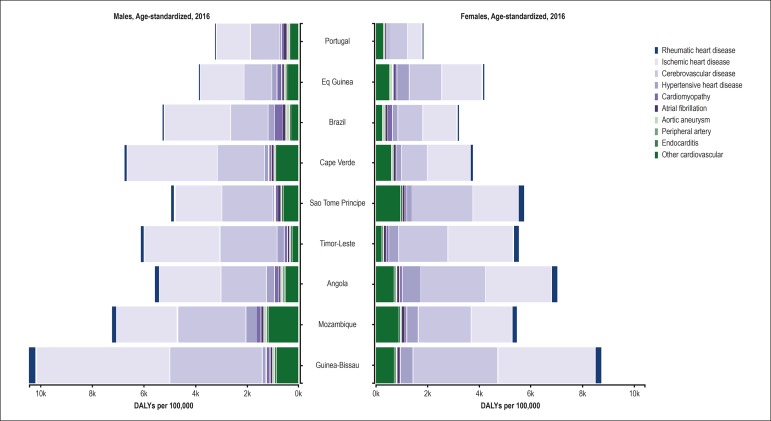
Disability-adjusted life years (DALYs) for each cardiovascular disease for each Portuguese-speaking country, 2016.

**Supplementary Figure 1:**
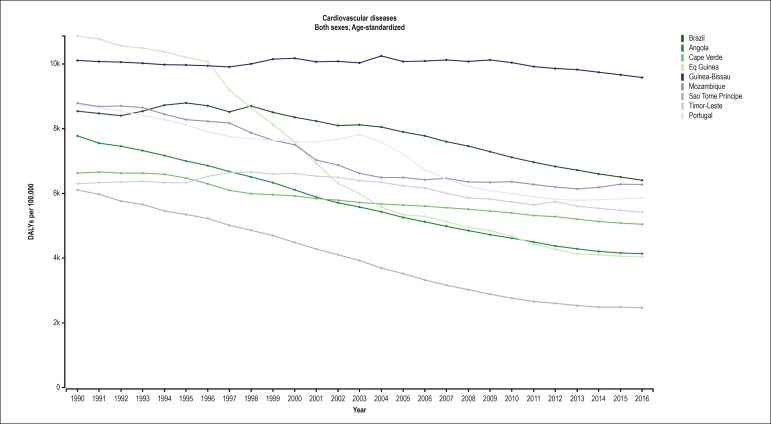
Age-standardized Disability Adjusted Life Years (DALY) attributable to cardiovascular disease (CVD) in Portuguese-speaking countries, from 1990 to 2016.

The trend in DALYs between 1990 and 2015 (Supplementary Figure 1) in the PSC is similar to that reported for the age-standardized mortality rate: there was a heterogeneous reduction in all countries, more expressive in those with better SDI. Regarding the specific causes of CVD, Figure 6 shows the importance of ischemic heart disease and CbVD in all countries, for both sexes. The loss of healthy life years was greater for men in all countries, except for Equatorial Guinea, Sao Tome and Principe and Angola, being mainly due to the other heart diseases. The importance of rheumatic heart disease, which is strictly related to socioeconomic conditions, for the loss of healthy years is evident in the countries with the lowest SDI.

### Influence of the risk factors on CVD

**Figure 7:**
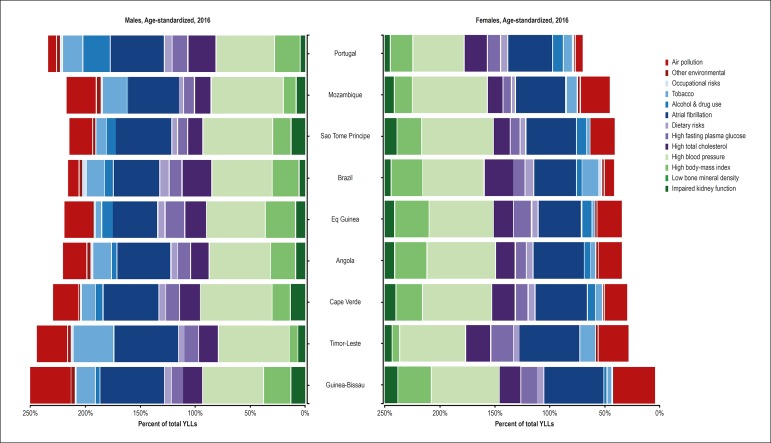
Influence of the risk factors on the years of life lost (YLLs) because of cardiovascular diseases, according to sex, in each Portuguese-speaking country in 2016.

Figure 7 reveals the risk factors attributed to the YLLs in each PSC. In general, of the classical risk factors and their determinants, arterial hypertension and dietary factors are the most important. The relevance of obesity is greater among women, being less important in Timor-Leste, despite the importance of metabolic risk factors in that country. The metabolic risk factors (high cholesterol, high blood sugar) have higher influence on the premature mortality from CVD in the countries with higher SDI (Portugal, Brazil and Equatorial Guinea). The relevance of the smoking habit is evenly greater for men, but heterogeneous among the countries. In addition, environmental risk factors, such as air pollution, are heterogeneous among the countries.

Detailed information about the metrics of the disease burden related to CVD and stratified by the PSC is shown in the Supplementary Tables.

## Discussion

Portuguese is the sixth most spoken language worldwide, by 244 million speakers,^16^ the fifth most commonly used language over the Internet, by 82.5 million cybernauts, and the third most commonly used language in the social media *Facebook* and *Twitter*, by 58.5 million people.^17^ According to estimates of the Portuguese government, considering the demographic evolution, by 2050, the number of Portuguese speakers will increase to 335 million. Africa should exceed Brazil in the use of Portuguese within 50 years.^16^

Portuguese is the official language of eight countries (Portugal, Brazil, Angola, Mozambique, Guinea-Bissau, Cape Verde, Sao Tome and Principe, and Timor-Leste). Despite the incorporation of native words and the grammatical and pronunciation changes characteristic of each country, their languages remain united to the Portuguese from Portugal, from which the PSC were colonies during the expansion of that nation. Independence from the Portuguese domination occurred at different points in time: Equatorial Guinea belonged to the Portuguese Empire only until 1778, being then given to Spain, while Brazil was a Portuguese colony until 1822. The other nations had their independence recognized by Portugal between 1973 and 1975. However, the cultural influence of Portugal on those countries has been striking, an example being Catholicism, the official religion in most of those countries, the predominance of the Portuguese surnames, in addition to the organization of the healthcare systems, which are similar in several aspects.^2,18^

Despite their cultural identity, the socioeconomic development was heterogeneous among the PSC. In 2015, the SDI was as low as 0.28 and 0.29 in Guinea-Bissau and Mozambique, respectively, although, in Brazil (0.66) and Equatorial Guinea (0.61), the SDI was closer to that of Portugal (0.75). The same occurred with the Gross Domestic Product per capita (GDP per capita), quantified in United States dollars in that same year, ranging from 606 to 611 in Guinea-Bissau and Mozambique, and from 20,247 to 21,619 in Equatorial Guinea and Portugal, respectively. The percentages of public expenditures on health differed, representing, in 2013, 2% to 3% of the GDP of most PSC, except for Portugal and Timor-Leste, whose percentages were higher, ranging from 9.1% to 10.4%, respectively, while Brazil was at an intermediate level, currently at 4% of the GDP.^2^

An important result of this study was to show that in all the PSC, except for Guinea-Bissau, reductions in age-standardized mortality rates from CVD were observed from 1990 to 2016. From a global perspective, the period was marked by a reduction in age-standardized mortality from CVD in all high-income and some middle-income countries, although little change could be observed in most Sub-Saharan countries.^7^ In the PSC, a strong positive correlation was observed between the SDI and the reduction in the age-standardized mortality rates from CVD in the past 27 years. However, that pattern was not homogeneous, suggesting that other factors^19^ could be associated with the observed mortality reduction.

In Brazil and Portugal, that correlation was closer. The decline in age-standardized mortality rates in Brazil, associated with the increase in life expectancy, and the maintenance of a proportional mortality around 30%, from 1990 to 2016, point towards a greater relevance of the reduction in the premature mortality component.^20^ The improvement in social inequality observed in Brazil and in some PSC might have contributed to that heterogeneous reduction in mortality as compared to the other Sub-Saharan countries.^21^

It is worth noting that, of the total number of deaths occurring in the Sub-Saharan Africa in 2015, ischemic heart disease was the fifth cause of death, preceded by the infectious causes in both sexes, while hemorrhagic stroke was the seventh, diabetes *mellitus*, the eighth, and ischemic stroke, the fifteenth cause of death in both sexes.^18^ In 2016, however, CVD were the first cause of death in almost all PSC, except for Guinea-Bissau, where CVD were the second cause of death, and Mozambique and Equatorial Guinea, where CVD were the third cause of death in both sexes. Ischemic heart diseases predominated in all PSC, except for Mozambique and Sao Tome and Principe, a different pattern from that observed in the other Sub-Saharan countries,^18^ suggesting a similarity between the PSC.

The DALYs were reduced in the PSC over the temporal series, from 1990 to 2016, probably reflecting an improvement in the health care provided to those populations.^22^ The DALYs were mainly due to ischemic heart disease and CbVD in those PSC. That reduction was greater in the countries with the highest SDI. However, in an analysis of the GBD Study from 1990 to 2013, the SDI did not explain the reduction in the DALYs due to CVD, mainly because of the heterogeneity of the set of countries considered.^22^

The systolic and diastolic blood pressure levels decreased from 1995 to 2015 in most high-income countries.^23^ That effect was not observed in most Sub-Saharan countries,^23^ and that could explain the predominance of the CbVD as the most important component of the mortality from CVD in Sub-Saharan countries, with the contribution of the increase in body mass and dietary factors.^24^ The same has occurred in the PSC, where arterial hypertension and dietary factors had a more relevant influence in both sexes for the DALYs due to CVD. It is worth noting the importance of the dietary risk factors in most PSC under the influence of the global dietary pattern, with ultra-processed foods and excessive amounts of sugars and fats, modifying healthier traditional dietary patterns. Those risk factors can be modified by promoting policies that favor healthy dietary habits, the taxation of ultra-processed foods, and the subsidies to healthy food, such as fruits and vegetables.^25,26^

The African populations are characterized by a great genetic diversity, representing the repository of genetic material of modern humans, who spread around the world over the past 100,000 years, having genetic adaptations in response to different climates, diets, geographic environments and infectious agents to which they have been exposed.^27^ The genetic variations of Sub-Saharan Africa have been modelled by geographical and ethnolinguistic similarities.^28^ In addition, in the European populations, linguistic similarities have shown to be better predictors of genome differences as compared to geographic differences.^29^ The complex genetic interactions with the environmental and sociodemographic factors will be able to help us understand the heterogeneous occurrence of CVD.^30^

Although the quality of the completeness of the data collected and estimates has varied between the different PSC, there was an improvement in the recent years of the temporal series,^4^ indicating the importance of the investment in national systems of vital registration and verbal autopsy to understand the burden of CVD in those countries.

### Limitations and strengths

The limitations of the analytical models of the GBD Study have been previously discussed in detail.^4-6,8^ Despite the improvement in the completeness of the data on prevalence and morbidity in some PSC, the estimates of the GBD 2016 Study indicate that the integrity and quality of those data are heterogeneous. For example, in Brazil and Portugal, the coverage of the data on death exceeds 95%, in contrast to very low or even absent indices in PSC of Sub-Saharan Africa.^4,8^ The GBD Study models may have been inadequate for the different countries in some groups of diseases, mainly the non-communicable ones and those under less strict epidemiological surveillance, such as the classification of Latin America as a non-endemic region for rheumatic heart disease, which differs from primary data of prevalence.^31,32^

In addition, the sociocultural, demographic, economic and ethnic differences and particularities between the PSC are not captured by the GBD Study models. Such differences are frequently associated with life habits, health behaviors and risk factors that affect the global burden of CVD. Moreover, despite having the same colonization and cultural similarities, the historical factors and development pattern of the societies differ significantly between the PSC.^2^

Despite those limitations, from the epidemiological point of view, the GBD Study is the most solid and comprehensive initiative to estimate the burden of CVD, being especially useful to enable standardized comparisons between countries, including the PSC, whose primary data are still scarce. Those limitations do not invalidate the importance of this study for the epidemiological evaluation of CVD in the PSC, aiming at the elaboration of educational, preventive and therapeutic strategies more adequate for each country's reality, considering their sociodemographic, economic and cultural differences.

The major strength of this analysis is to consistently demonstrate that the importance of the CVD as a cause of death has grown in the PSC. Although mortality has decreased or remained stable in countries with better SDI - with a significant reduction in age-standardized mortality rates - the same pattern has not been observed in the countries with worse SDI, indicating the important impact of CVD and their association with socioeconomic factors. The GBD Study estimates have great relevance for the continuous reassessment of policies of prevention and health promotion, as well as for the formulation, planning and adequacy of new strategies to be implemented. Regional trends of morbidity and premature mortality from certain groups of CVD, especially in countries with lower SDI and less-structured health systems, should be considered, aiming at the individualization of action plans for countries with similar cultural origin, but very different health realities.

## Conclusion

The data presented show great differences in the relative importance of the burden of CVD in the PSC and indicate that such differences relate to the socioeconomic conditions of the countries. Of the CVD, ischemic heart disease is the major cause of death in all PSC, except for Mozambique and Sao Tome and Principe, where CbVD is the major cause. The PSC share the most relevant risk factors for CVD: hypertension and dietary factors. Genetic factors, implicit in the cultural identity, factors inherent in the host, as well as the huge social inequality might have contributed to explain the mortality rates observed. Collaboration between the PSC might allow sharing successful experiences to confront CVD between those countries.

## Figures and Tables

**Supplementary Figure 2:**
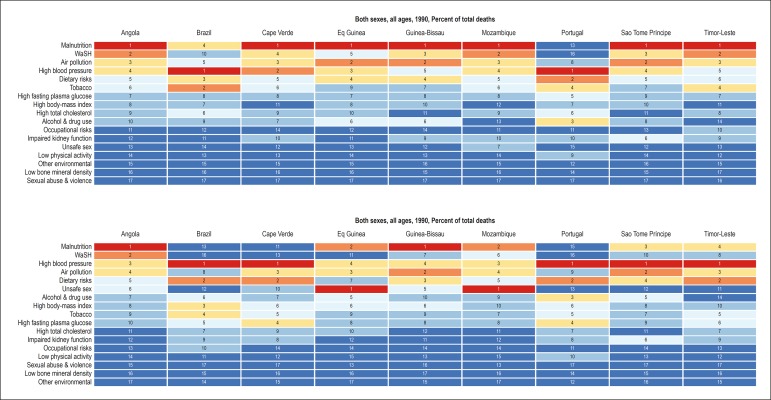
Mortality attibutable to risk factors in Portuguese-speaking countries, in 1990 and 2016.
